# Genetic diversity of non-*Saccharomyces* yeasts associated with spontaneous fermentation of Cabernet Sauvignon wines from Ningxia, China

**DOI:** 10.3389/fmicb.2023.1253969

**Published:** 2023-08-17

**Authors:** Ruirui Li, Danping Feng, Hui Wang, Zhong Zhang, Na Li, Yue Sun

**Affiliations:** ^1^School of Food Science and Engineering, Ningxia University, Yinchuan, Ningxia, China; ^2^College of Life Sciences, Northwest A and F University, Yangling, Shaanxi, China; ^3^School of Life Sciences, Ningxia University, Yinchuan, Ningxia, China; ^4^College of Enology and Horticulture, Ningxia University, Yinchuan, Ningxia, China; ^5^Wine Institution of Ningxia Region, Yinchuan, Ningxia, China

**Keywords:** non-*Saccharomyces*, diversity, wine fermentation, molecular fingerprinting, TRtRNA

## Abstract

The organoleptic profile and quality of wine are affected by the presence of different non-*Saccharomyce*s species and strains. Therefore, the identification and characterization of non-*Saccharomyce*s yeasts are the first step to understand their function, and to develop a better strain selection program for winemaking. This study investigated the biodiversity of non-*Saccharomyces* yeasts associated with spontaneous fermentation of Cabernet Sauvignon wines from five sub-regions (Shi Zuishan, Yinchuan, Yu Quanying, Qing Tongxia and Hong Sibu) in Ningxia, China. Yeast species were identified by sequencing the 26S rRNA D1/D2 region, and strains at the subspecies level were discriminated using tandem repeat-tRNA (TRtRNA) PCR analysis. A total of 524 yeast colonies were isolated, and 19 non-*Saccharomyces* yeast species belonging to 10 genera were identified, including *Aureobasidium pullulans*, *Cryptococcus albidus*, *Cryptococcus* sp.*, C. flavescens*, *C. terrestris*, *C. magnus*, *Cystofilobasidium ferigula*, *Candida zemplinina*, *Filobasidium magnum*, *Filobasidium* sp.*, F. elegans*, *Hanseniaspora uvarum*, *Metschnikowia pimensis*, *M. pulcherrima*, *Naganishia albida*, *Pichia kluyveri*, *P. kudriavzevii*, *Rhodotorula glutinis* and *R. graminis*. *Hanseniaspora uvarum*, *C. zemplinina*, and *M. pulcherrima* were the three most dominated species, while other non-*Saccharomyces* species were only present in the early stage of spontaneous fermentations at different levels. Further, for the yeast discrimination at strain level, 34 profiles were obtained by amplification with primer pairs TtRNASC/5CAG, while 40 profiles were obtained with primer pairs TtRNASC/ISSR-MB. This study explored the diversity of non-*Saccharomyces* species in Ningxia, China, and made an important contribution of genetic resources for further strain development.

## 1. Introduction

During wine fermentation, yeasts could convert sugar to ethanol and carbon dioxide, glycerol, higher alcohols, aldehydes and esters, etc. According to the strength of fermentation capacity, yeasts are classified as *Saccharomyces cerevisiae* and non*-Saccharomyces*. In the past, non*-Saccharomyces* yeasts were considered as undesired or spoilage yeasts during wine fermentation. However, recent studies have showed the positive contributions of non*-Saccharomyces* yeasts to wine quality, such as generating aromatic compounds through enzymatic reactions, increasing glycerol production, decreasing ethanol content and stabilizing wine color (Ciani et al., 2016; Padilla et al., 2016; Medina et al., 2017; Benito et al., 2019; Morata et al., 2020; Maicas and Mateo, 2023). Moreover, most of these enological characteristics are species and strain dependent (Padilla et al., 2016; Binati et al., 2019). Therefore, the discrimination of different non*-Saccharomyces* yeast strains is a step of primary importance to their biotechnological application. In recent years, the regional flavor peculiarity of non*-Saccharomyces* strains has drawn widespread attention, and there are increasing demands for wine style diversification and local characteristics. Therefore, growing studies worldwide begin to focus on the development and utilization of wine-related non*-Saccharomyces* yeasts (Binati et al., 2019; Zhao et al., 2021; Lai et al., 2022; Morata et al., 2022; Tamang and Lama, 2023).

The research on the isolation, identification and enological characteristics of non*-Saccharomyces* yeasts is one of the research hot spots at present, which provides a reliable reference for the exploring of local non*-Saccharomyces* strains with great industrial potential (Casas-Godoy et al., 2021). Considering the great diversity and potential applications of different non-*Saccharomyces* yeast strains within the same species, it is important to discriminate them at both species and subspecies level for subsequent study. Although there are several well documented molecular biological techniques for identifying yeast species such as sequencing of 26S rRNA D1/D2 (Kurtzman and Robnett, 1998) and restriction analysis of the 5.8S rDNA ITS region (Guillamón et al., 1998; Lorca et al., 2018), there are limited studies available for discriminating non-*Saccharomyces* yeasts at subspecies level. In recent years, various technologies of molecular fingerprinting have been used for distinguishing non*-Saccharomyces* strains, such as random amplified polymorphic DNA (RAPD) (Binati et al., 2019), fourier transform infrared spectroscopy (FTIR) (Nieuwoudt et al., 2006; Grangeteau et al., 2016), tandem repeat-tRNA (TRtRNA) (Barquet et al., 2012), amplified fragment length polymorphisms (AFLP) (Esteve-Zarzoso et al., 2010; Albertin et al., 2016; Baselga et al., 2017), and microsatellites (Albertin et al., 2016). However, most of these methods have the drawbacks of laborious procedures and are not suitable for handling large quantity of samples. In addition, RAPD technology is susceptible to various factors, such as Mg^2+^, dNTP, template, and reaction temperature. FTIR cannot complete qualitative and quantitative identification of the cell components in a practical situation due to the effects of environmental changes. AFLP data are difficult to interpret, and requires complex bioinformatic programs. Compared with those methods, TRtRNA-PCR offers simplicity, reproducibility, and high discrimination power for typing non-*Saccharomyces* yeast strains (Barquet et al., 2012). According to the trend of simplification and automation, TRtRNA is a promising method to discriminate non-*Saccharomyces* yeast strains.

**Figure 1:**
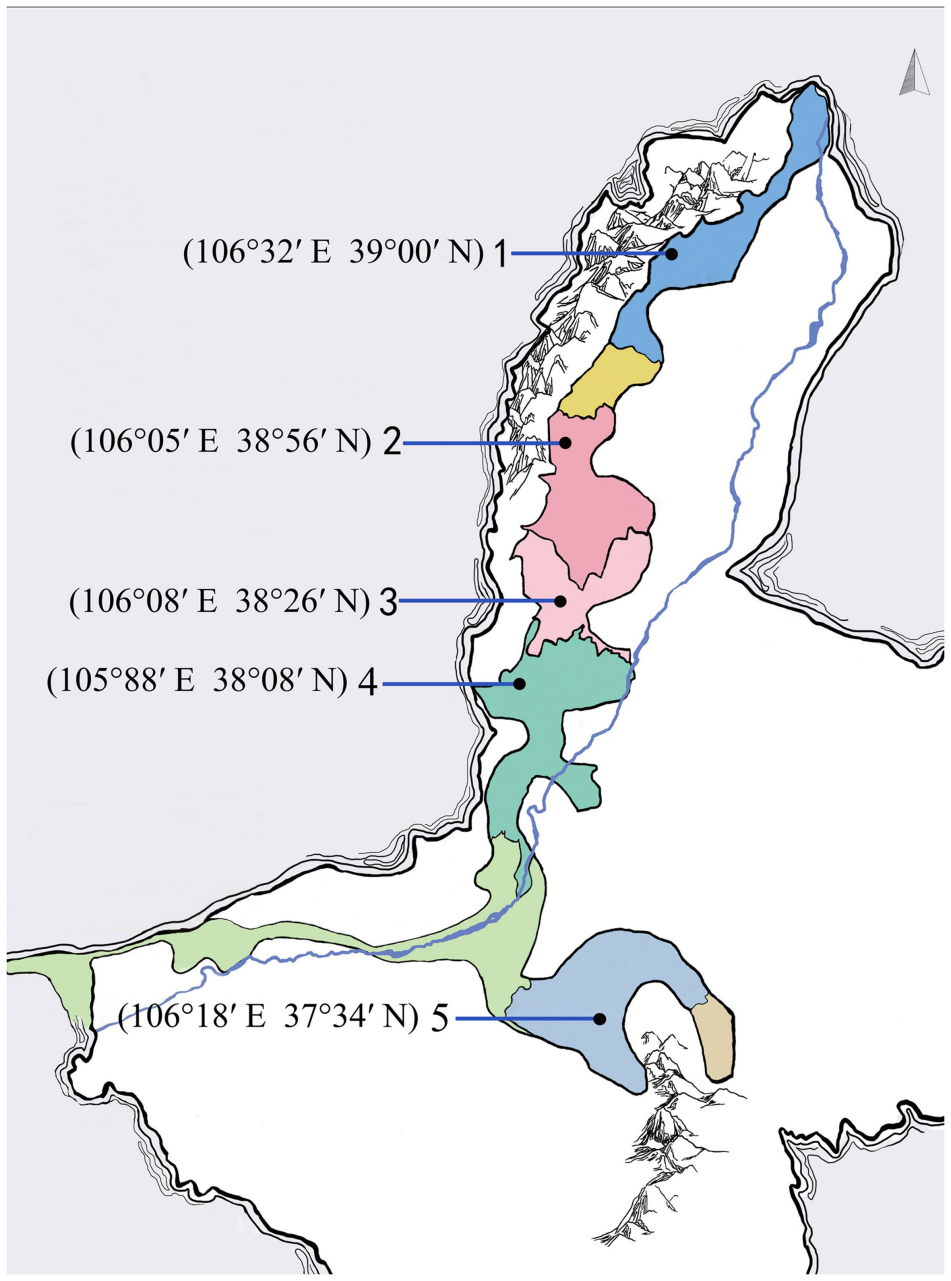
The geographic information of sampling points from five sub-regions in Ningxia, China.

Ningxia is one of the oldest and promising wine producing regions in China (Sun et al., 2017). It can be divided into five sub-regions named Shi Zuishan, Yinchuan, Yu Quanying, Qing Tongxia and Hong Sibu (Figure 1), according to their different geographical location and climatic condition (Li et al., 2022). The typical geographical and climatic characteristics have facilitated to form rich and diverse yeast communities. In recent years, several case studies have explored the density and diversity of indigenous yeast community in Ningxia. Yeast species identified in Ningxia are generally associated with genera *Candida*, *Hanseniaspora*, *Issatchenkia*, *Metschnikowia, Pichia*, *Saccharomyces*, *Torulaspora* and *Zygosaccharomyces* (Li et al., 2011; Sun et al., 2017; Zhang et al., 2020). Although the enological characteristics of a few non*-Saccharomyces* yeast species were investigated, the wealth of non*-Saccharomyces* yeast biodiversity with still hidden potential in wine fermentation needs further investigation.

The aims of the present work were to evaluate the genetic diversity and relatedness among non-*Saccharomyces* strains of five sub-regions of Ningxia, to establish a strain collection to preserve the non-*Saccharomyces* genetic resources of China, and to provide reference and scientific basis for their further development for regional wine production. Firstly, this study analyzed the phylogenetic diversity of indigenous non-*Saccharomyces* yeasts isolated from Cabernet Sauvignon spontaneous fermentations. Then, the dynamics of different non-*Saccharomyces* species associated with fermentation stage and sub-regions were evaluated. Finally, TRtRNA-PCR was used to discriminate non-*Saccharomyce*s yeasts at subspecies level. Dendrograms were constructed based on similarity among different patterns of bands, and the genetic relationships of all strains were also evaluated.

## 2. Materials and methods

### 2.1. Isolation of indigenous non-*Saccharomyces* yeast strains

The Cabernet Sauvignon grapes for this study were harvested in 5 sub-areas of Eastern Foot of Helan Mountain named Shi Zuishan, Yinchuan, Yu Quanying, Qing Tongxia and Hong Sibu, Ningxia, China (Figure 1). In 2020, Ripe and physically undamaged grapes were manually destemmed and crushed, and naturally fermented with the skin under aseptic conditions. The initial sugar content, total acidity, pH of grapes and residual sugars, alcohol content of final wines were shown in Supplementary Table S1. All the fermentations were conducted in 10.0 L fermenters, and the fermentations were performed in duplicate. 60 mg/L of potassium metabisulfite and 20 mg/L of pectinase were added to the must after crushing. The fermentation temperature was controlled at 25–28°C. The progress of the fermentations was monitored by measuring CO_2_ loss every 12 h (Supplementary Figure S1). Samples were collected during spontaneous fermentations at 1 d (grape must after crushing), 2 d, 3 d and 5 d.

At each stage, aliquots of several dilutions (from 10^−1^ to 10^−6^) were spread in triplicate on WLN agar (Hopebiol, China) and supplemented with 100 mg/L chloramphenicol to inhibit bacterial growth (Pallmann et al., 2001). For each sample, based on the colony morphology and frequency, 15 to 25 non-*Saccharomyces* colonies were proportionately isolated from the selected dilution plate, thus yielding 524 isolates for the subsequent identification. After purification on YPD agar (containing 2% glucose, 2% peptone, and 1% w/v yeast extract). All the strains were stored in 20% glycerol at −80°C for subsequent identification.

### 2.2. DNA extraction

The DNA extraction protocol for Non-*Saccharomyces* yeasts was adapted from previous studies (Renouf et al., 2007; Sun and Liu, 2014). 19 species in 10 genera of yeasts, *Aureobasidium pullulans*, *Cryptococcus albidus*, *Cryptococcus* sp.*, C. flavescens*, *C. terrestris*, *C. magnus*, *Cystofilobasidium ferigula*, *Candida zemplinina*, *Filobasidium magnum*, *Filobasidium* sp.*, F. elegans*, *Hanseniaspora uvarum*, *Metschnikowia pimensis*, *M. pulcherrima*, *Naganishia albida*, *Pichia kluyveri*, *P. kudriavzevii*, *Rhodotorula glutinis* and *R. graminis* were included. Briefly, the biomass grown on Petri dishes were collected in TE buffer and washed once. Then, 500 μL of nuclei lysis (Promega) and 300 μL of protein precipitation solution (Promega) were used for the DNA extraction. Residual polyphenols were precipitated after addition of 100 μL of a 10% polyvinyl-pyrrolidone (PVP; Sigma-Aldrich). Visible mass of DNA was obtained by addition 500 μL of isopropanol, and then 70% ethanol solution was added to re-suspend the pellet. Finally, DNA was qualified using DN-1000 Spectro-photomter (NanoDrop, United States). All DNA samples were stored at −20°C before use.

### 2.3. Identification of non-*Saccharomyces* species

All 524 yeast isolates were identified by sequence analysis of the 26S rRNA D1/D2 domain with primers NL1 and NL4 according to Kurtzman and Robnett (1998). PCR was performed in a final volume of 25 μL containing 0.2 mM of each dNTP, 1 μL DNA template, 1.5 mM MgCl_2_, 0.2 μM of each primer, 0.1 U Easy Taq Buffer Polymerase (TransGen Biotechnology, Beijing, China) and 1 × Easy Taq Buffer. Samples were subjected to the following thermal profile for amplification in FlexCycler2 PCR Thermal Cyclers (Model 844–00069, Analytik Jena AG, Jena, Germany): initial denaturation at 95°C for 5 min, 36 cycles of 94°C for 1 min, 52°C for 1 min and 72°C for 80 s, and a final extension at 72°C for 8 min. PCR products were separated by 2% (w/v) agarose gel electrophoresis in 0.5 × TBE buffer for 40 min at 110 V. The products gave positive results were sent to Sangon Biotech (Shanghai) Co. Ltd. for purification and sequencing. The sequences were analyzed using the Blast method of NCBI.^1^ DNA sequences were deposited in GenBank under the accession numbers: OP445815 *Aureobasidium pullulans* (NXU 21–01), OP445816 *Cryptococcus albidus* (NXU 21–02), OQ857299 *Cryptococcus* sp. (NXU 21–18), OP445817 *Cryptococcus flavescens* (NXU 21–03), OP445818 *Cryptococcus terrestris* (NXU 21–04), OP445819 *Cryptococcus magnuse* (NXU 21–05), OP445820 *Cystofilobasidium ferigula* (NXU 21–06), OP445821 *Candida zemplinina* (NXU 21–07), OP445822 *Filobasidium magnum* (NXU 21–08), OQ857300 *Filobasidium* sp. (NXU 21–19), OP445823 *Filobasidium elegans* (NXU 21–09), OP445824 *Hanseniaspora uvarum* (NXU 21–10), OP445825 *Metschnikowia pimensis* (NXU 21–11), OP445826 *Metschnikowia pulcherrima* (NXU 21–12), OP445827 *Naganishia albida* (NXU 21–13), OP445828 *Pichia kluyveris* (NXU 21–14), OP445829 *Pichia kudriavzevii* (NXU 21–15), OP445830 *Rhodotorula glutinis* (NXU 21–16) and OP445831 *Rhodotorula graminis* (NXU 21–17).

### 2.4. Phylogenetic analysis

The sequences of different non-*Saccharomyces* species were aligned using MAFFT version 7 (Katoh and Standley, 2013), and the sequence alignment was subjected to Maximum Likelihood analysis using IQ-TREE version 2.2.0 (Minh et al., 2020). Bootstrap values were calculated from 1,000 iterations.

### 2.5. Tandem repeat-tRNA PCR analysis

Tandem repeat-tRNA fingerprinting (TRtRNA) analysis of all non-*Saccharomyces* strains was carried out with TtRNASc (5′- GCTTCTATGGCCAAGTTG-3′), ISSR-MB (5′-CTCACAACAACAACAACA-3′), and 5CAG (5′- CAGCAGCAGCAGCAG-3′) according to Barquet et al. (2012). Amplification was performed as follows: volume of 20 μL, 2.5 mM MgCl_2_, 0.2 mM dNTPs, 1 μM each primer, 2 uL 10 × Easy Buffer and 1 U Taq DNA polymerase and 1 μL genomic DNA. Amplification was procedure as follows: Predenaturation 95°C for 5 min; Denaturation at 95°C for 60s and annealing at 50°C for 60s, 35 cycles of 90s at 72°C, and a final extension of 10 min at 72°C. The PCR products were separated by electrophoresis on 1.2% agarose gels which was applied with 100 V for 45 min in 0.5 × TBE buffer and photographed under UV light. TRtRNA sequence types of Angel yeast NS-D (*T. delbrueckii*), Excellence B-Nature (*M. pulcherrima*) and Excellence X-Fresh (*L. thermotolerans*) were used as references to compare the genetic profiles of the tested non-*Saccharomyces* strains.

### 2.6. Cluster analysis of the strains

The TRtRNA sequence patterns obtained after gel electrophoresis were used for the construction of a presence/absence matrix, taking into account the total number of different bands observed (Sun et al., 2017). Similarities based on the Dice coefficient were calculated and UPGMA clustering was obtained using NTSYS software (Mercado et al., 2010).

## 3. Results

### 3.1. Phylogenetic analysis of yeast isolates from different sub-regions of Ningxia

A total of 524 non-*Saccharomyces* isolates were collected during spontaneous fermentation of Cabernet Sauvignon wines from 5 sub-regins of Ningxia, China. As showed in Table 1, 19 species in 10 genera of non-*Saccharomyces* yeasts were identified by sequencing the 26S rRNA D1/D2 domain. The identified yeast species include *Aureobasidium pullulans*, *Cryptococcus albidus*, *Cryptococcus* sp.*, C. flavescens*, *C. terrestris*, *C. magnus*, *Cystofilobasidium ferigula*, *Candida zemplinina*, *Filobasidium magnum*, *Filobasidium* sp.*, F. elegans*, *Hanseniaspora uvarum*, *Metschnikowia pimensis*, *M. pulcherrima*, *Naganishia albida*, *Pichia kluyveri*, *P. kudriavzevii*, *Rhodotorula glutinis* and *R. graminis.* Table 1 shows the distribution, and origin of these yeast species. Regarding the different yeast species*, H. uvarum and C. zemplinina* were the two most abundant species. The proportion of *H. uvarum* was 73.66% (386/524) followed by *C. zemplinina,* 11.26% (59/524), while the proportions of other 17 species were less than 4.20% (22/524).

**Table 1:** The yeast species and their frequency isolated from spontaneous fermentations of Cabernet Sauvignon wines in different sub-regions.

Species	Number of isolates/Colony frequency (%)						Representative isolates	Accession numbers
	Shizuishan	Yinchuan	Yuquanyin	Qingtongxia	Hongsipu	Total		
*Hanseniaspora uvarum*	78 (67.82)	81 (71.68)	81 (85.26)	59 (67.05)	87 (76.99)	386 (73.66)	NXU 21–10	OP445824
*Candida zemplinina*	11 (9.57)	7 (6.19)	8 (8.42)	13 (14.77)	20 (17.70)	59 (11.26)	NXU 21–07	OP445821
*Metschnikowia pulcherrima*	1 (0.87)	13 (11.50)	6 (6.32)	2 (2.27)	−	22 (4.20)	NXU 21–12	OP445826
*Cryptococcus* sp.	13 (11.30)	−	−	1 (1.14)	−	14 (2.67)	NXU 21–18	OQ857299
*Filobasidium magnum*	−	5 (4.42)	−	7 (7.95)	−	12 (2.29)	NXU 21–08	OP445822
*Cryptococcus albidus*	4 (3.48)	1 (0.88)	−	2 (2.27)	−	7 (1.34)	NXU 21–02	OP445816
*Pichia kluyveri*	−	2 (1.77)	−	−	2 (1.77)	4 (0.76)	NXU 21–14	OP445828
*Pichia kudriavzevii*	−	−	−	−	4 (3.54)	4 (0.76)	NXU 21–15	OP445829
*Cystofilobasidium ferigula*	3 (2.61)	−	−	−	−	3 (0.76)	NXU 21–06	OP445820
*Rhodotorula graminis*	−	2 (1.77)	−	−	−	2 (0.38)	NXU 21–17	OP445831
*Aureobasidium pullulans*	−	2 (1.77)	−	−	−	2 (0.38)	NXU 21–01	OP445815
*Cryptococcus flavescens*	1 (0.87)	−	−	1 (1.14)	−	2 (0.38)	NXU 21–03	OP445817
*Naganishia albida*	1 (0.87)	−	−	−	−	1 (0.19)	NXU 21–13	OP445827
*Filobasidium* sp.	−	−	−	1 (1.14)	−	1 (0.19)	NXU 21–19	OQ857300
*Metschnikowia pimensis*	1 (087)	−	−	−	−	1 (0.19)	NXU 21–11	OP445825
*Filobasidium elegans*	−	−	−	1 (1.14)	−	1 (0.19)	NXU 21–09	OP445823
*Cryptococcus terrestris*	1 (0.87)	−	−	−	−	1 (0.19)	NXU 21–04	OP445818
*Cryptococcus magnus*	−	−	−	1 (1.14)	−	1 (0.19)	NXU 21–05	OP445819
*Rhodotorula glutinis*	1 (0.87)	−	−	−	−	1 (0.19)	NXU 21–16	OP445830
Total	115	113	95	88	113	524		

‘−’ not detected.

**Figure 2:**
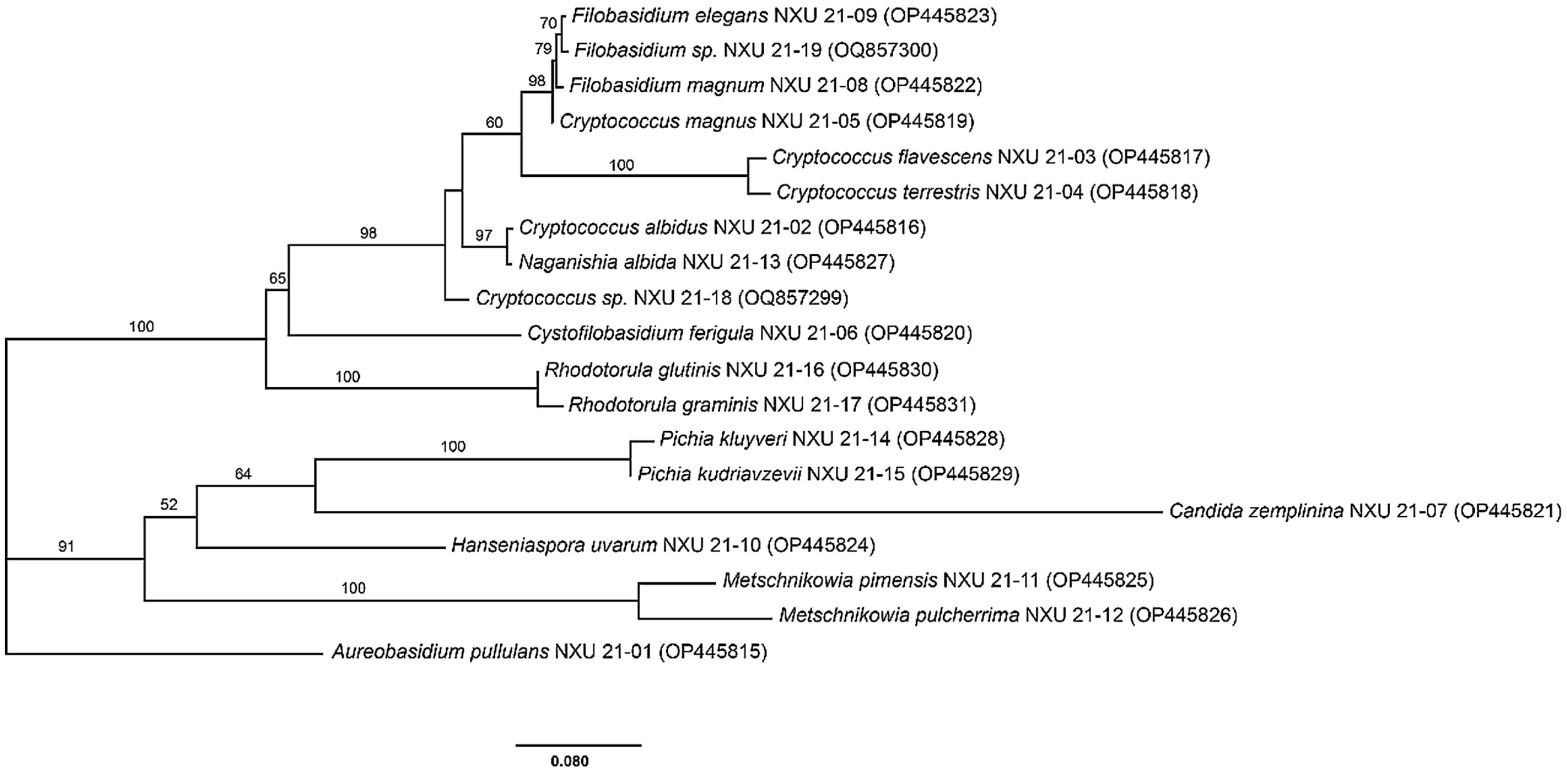
Phylogenetic tree of representative non-*Saccharomyces* yeast isolates obtained from five sub-regions of Ningxia based on the sequence analysis of the 26S rRNA D1/D2 region using the Maximum-Likelihood method. The scale bar shows 0.080, Bootstrap support values were estimated based on 1,000 replicates and are showed above the branches (> 50%).

To evaluate the evolutionary relationship of these species, a phylogenetic tree reflecting the genome homology and genetic distance of the yeasts was constructed (Figure 2). It is evident from the phylogenetic tree that *A. pullulans* formed a single clade while the other 18 species showing two main clades. In the phylogenetic tree, *C. flavescens* and *C. terrestris, R. glutinis* and *R. graminis, P. kluyveri* and *P. kudriavzevii, M. pimensis and M. pulcherrima* were clustered together, respectively.

### 3.2. Dynamics of non*-Saccharomyces* species during fermentations in different sub-regions

**Figure 3:**
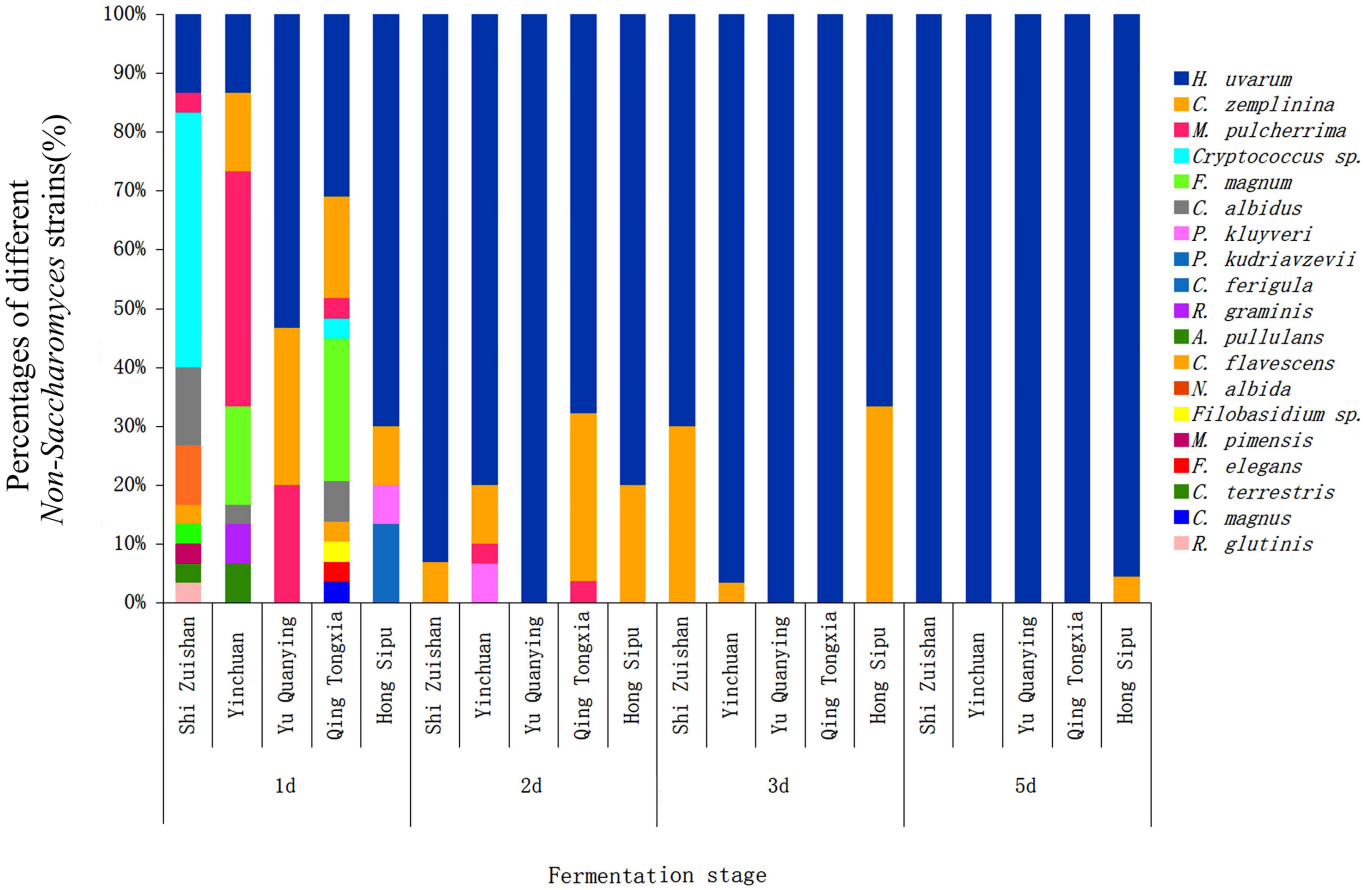
The relative abundance of non-*Saccharomyces* species isolated from different stages during Cabernet Sauvignon spontaneous fermentation in Shi Zuishan, Yin Chuan, Yu Quanying, Qing Tongxia, Hong Sibu.

To explore the dynamic changes of non-*Saccharomyces* species during the spontaneous fermentation of Cabernet Sauvignon in different sub-regions, the numbers of yeasts at 1 d, 2 d, 3 d and 5 d were analyzed in this study. The yeast diversity and community changes at the species level during spontaneous fermentations are showed in Figure 3. An obvious change of yeast community composition was observed during the whole process in all five sub-regions. In general, day 1 showed the highest yeast diversity, and the numbers of yeast species decreased during fermentation process. More specifically, the decrement of yeast species in Shi Zuishan and Qing Tongxia was particularly evident, which decreased from 10 species at day 1 to 2 and 3 species, respectively, at day 2. However, the lowest yeast diversity was found in Yu Quanying with 3 species of *H. uvarum, C. zemplinina* and *M. pulcherrima* at day 1. On day 5, only Hong Sibu could be isolated 2 different yeast species compared with 1 species isolated in other four sub-regions. It was noted that *H. uvarum* showed an increasing trend throughout the fermentation process, especially in Yu Quanying, and the proportion of *H. uvarum* achieved up to 100% on day 2 until day 5. *C. zemplinina* was the secondly abundant species on day 2 to day 5. *N. albida*, *R. glutinis*, *C. terrestris*, *C. ferigula* and *M. pimensis* were only found in Shi Zuishan and were not detected in the other four sub-regions.

### 3.3. Intraspecific genetic diversity in non*-Saccharomyces*

**Figure 4:**
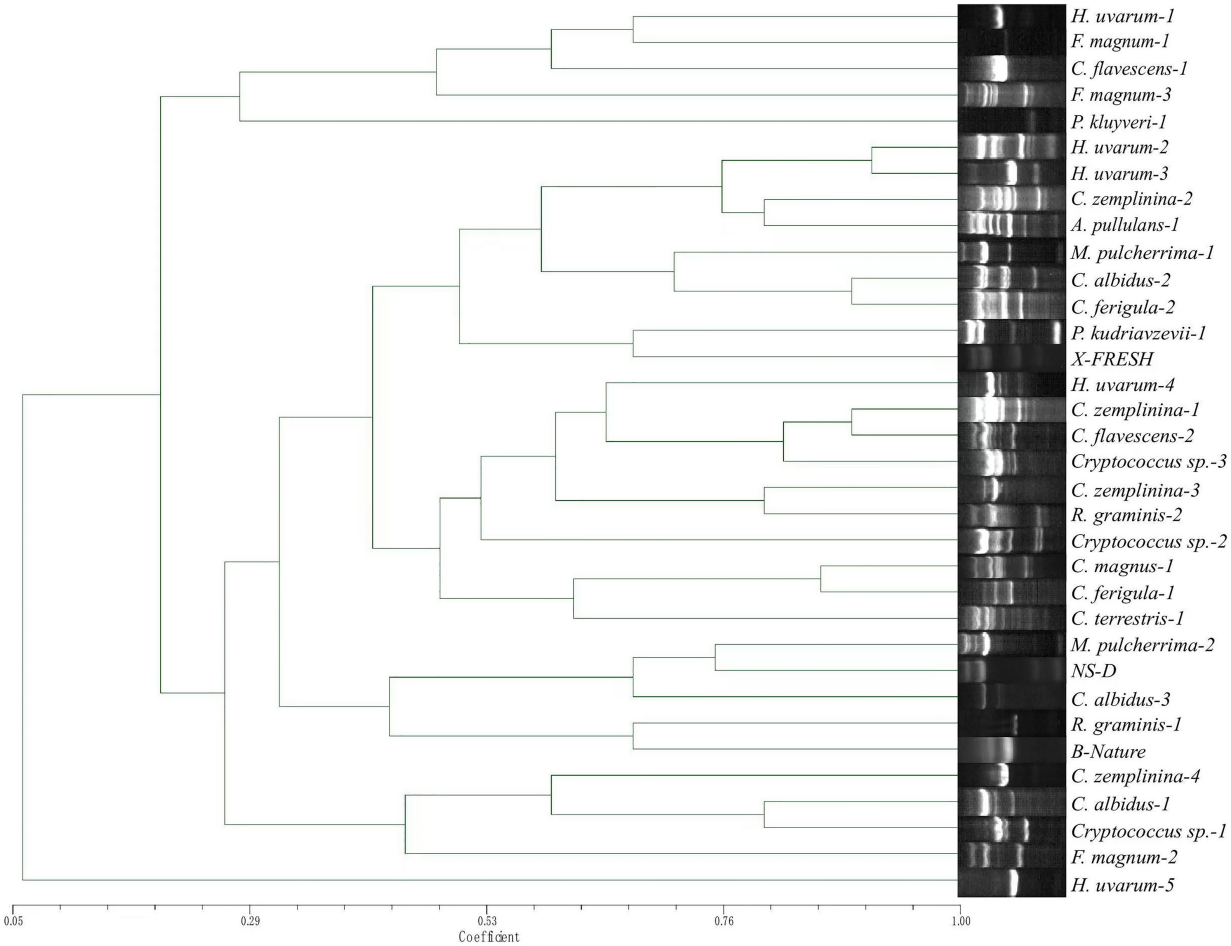
Unweighted pair group method with arithmetic mean dendrogram showing genetic relatedness of the non-*Saccharomyces* isolates obtained from Eastern foot of Helan Mountain distinguished by primer pairs TRtNASC and 5CAG.

**Figure 5:**
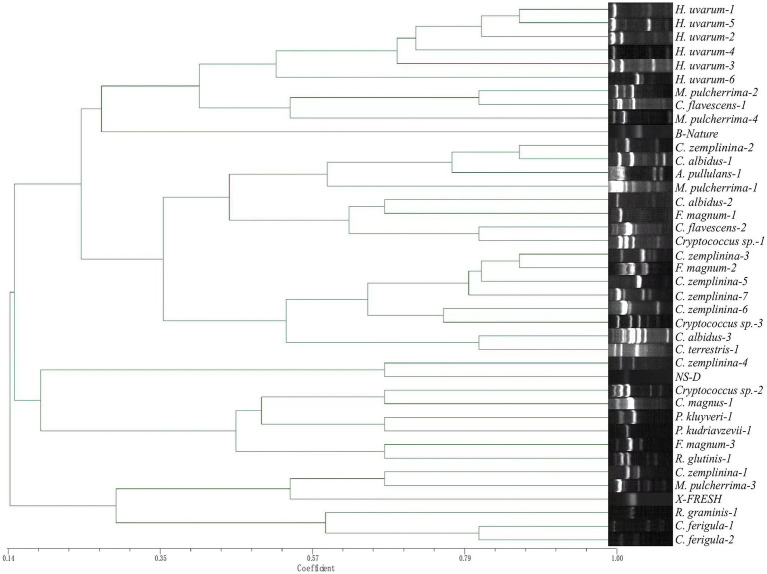
Unweighted pair group method with arithmetic mean dendrogram showing genetic relatedness of the non-*Saccharomyces* isolates obtained from Eastern foot of Helan Mountain distinguished by primer pairs TRtNASC and ISSR-MB.

TRtNASC/5CAG and TRtNASC/ISSR-MB discriminated the 524 non-*Saccharomyces* isolates into 34 (Figure 4) and 40 profiles (Figure 5), respectively. Among them, neither primer pair could discriminate *F. elegans*, *Filobasidium* sp., *M. pimensis* and *N. albida*. Compared with TRtNASC/5CAG, pairs of primers TRtNASC/ISSR-MB resulted in a higher discrimination power with the exception of *R. graminis.* More specifically, TRtNASC/ISSR-MB could discriminate *H. uvarum* (386 isolates), *C. zemplinina* (59 isolates), *M. pulcherrima* (22 isolates), *Cryptococcus sp.*, (14 isolates), and *R. glutinis* (1 isolate) into 6, 7, 4, 3 and 1 profiles, respectively (Figure 5). However, TRtNASC/5CAG could discriminate these yeast species into 5, 4, 2, 2 and 0 profiles, respectively (Figure 4). For the other 9 yeast species, both the two pairs showed the same discrimination power for the same species.

Genetic relatedness was also evaluated by constructing dendrograms compiled from all Tandem repeat-tRNA (TRtRNA) fingerprinting. For primer pairs TRtNASC and ISSR-MB (Figure 5), an obvious difference was observed in the relationships of different non-*Saccharomyces* species. All 8 strains of *H. uvarum* were clustered together when the Dice coefficient is 0.482. *Cystofilobasidium ferigula formed a single clade,* while most of the *Cryptococcus* species except for *Cryptococcus sp.*-2, *C. magnus*-1 and *C. zemplinina* were cluster together with a Dice coefficient of 0.35. Six groups are indicated in Figure 4, when the Dice coefficient is 0.434. *Pichia kudriavzevii*-1 *and H. uvarum-5 formed a single clade respectively,* while other groups contain at least four species.

## 4. Discussion

Non-*Saccharomyces* yeasts are essential in the development of important metabolites that determine the wine flavor and aroma, greatly influencing the characteristics of wine (Jolly et al., 2013). Understanding the genetic diversity of non-*Saccharomyces* strains can make an important contribution toward delineating the genetic distance of these strains, as well as providing genetic material for further strain development.

### 4.1. Non*-Saccharomyces* yeast species and their function

In this study, the genetic diversity of indigenous non-*Saccharomyces* strains was investigated during the spontaneous fermentations of Cabernet Sauvignon grown in 5 sub-areas of Ningxia wine region, China. 19 yeast species in 10 genera were isolated and identified in this study. Compared to previous studies (Li et al., 2011; Sun et al., 2017; Wang et al., 2017; Zhang et al., 2020, 2021), the yeast species of *C. zemplinina*, *H. uvarum, M. pulcherrima* and *P. kluyveri* were the frequently found species but with different distribution. However, some of these species like *A. pullulans*, *C. albidus, C. flavescens, C. terrestris, C. ferigula, F. magnum, F. elegans, F. magnum, M.pimensis, N. albida, R. glutinis* and *R. graminis* have not been characterized previously in this region. The findings from these different studies suggest the time- and location-dependent variations in yeast communities. In addition, this study revealed that in different sub-regions the yeast distributions were different. Specifically, 10 species in 7 genera were found in Shizuishan compared to 3 species in 3 genera found in Yuquanying. The same observations were made in the Shangri-La wine region, China (Zhao et al., 2021), which showed that different non-*Saccharomyces* associated with spontaneous fermentation of Cabernet Sauvignon were present at different geographical location with different microclimate characteristics. Likewise, in our study, differences in location and microclimate were responsible for the large effect on the variation in yeast diversity during spontaneous fermentation of Cabernet Sauvignon wines in different sub-regions. Since yeast community variations during spontaneous fermentation are attributed to the location, vintage year, cultivar., enological practices (such as SO_2_ addition and yeast inoculation) (Andorrà et al., 2008; Sun and Liu, 2014; Wang et al., 2021), our findings represent only a ‘snapshot in time’. Garofalo et al. (2016) found significant differences in non-*Saccharomyces* biodiversity among locations and vintages. Consequently, in order to obtain a more reliable result of non-*Saccharomyces* yeast community, more research on yeast isolation and preservation should be performed consistently for long periods of time.

Recently, the enological behaviors and potential application of indigenous non-*Saccharomyces* have drawn widespread attention (Zhao et al., 2022; Castrillo and Blanco, 2023). Although *H. uvarum*, *M. pulcherrima* and *C. zemplinina* are the abundant yeast species during wine fermentation in this study. The function of minor species such as *N. albida* and *F. magnum* were also of special interest in our study, and their current use in winemaking applications is rather limited*. N. albida* and *F. magnum* were the dominant species isolated from Qaidam Basin Desert, China, with Mars-like extreme environments such as hyper-arid, salt deposits, high UV, similar mineral compositions and so on (Wei et al., 2022). In our study, *F. magnum* maintained a larger population at day 1 in YinChuan (16.67%) and Qing Tongxia (24.14%). Generally, Ningxia belongs to arid and semiarid climate with saline soil. It is understandable that this environment has contributed to their occurrence in the grape must (day 1). *F. magnum* was found as an endophytic fungus in the vineyard environment and was associated with the formation of grape flavor (Sayed et al., 2021), making it a candidate for enhancing wine flavor in our future study of yeast selection program. Other yeasts like *A. pullulans, C. albidus* and *C. magnus* possess biological control activity against fungal pathogen of grape and other fruits (De Capdeville et al., 2007; Pantelides et al., 2015; Türkoğlu et al., 2022). These yeasts have the potential to suppress phytopathogens by competition for space and the production of substances that affect hyphae integrity (niche competition). In addition, species having potential roles in wine aroma, flavor and texture during the early stages of fermentation and finished wines were of special interest in our study. Perhaps of most importance to Ningxia wine makers, *R. graminis* and *R. glutinis* has the potential as a flavor producer due to their lipid production (Martinez-Silveira et al., 2022; Zhao and Li, 2022). Future studies will investigate their potential in winemaking.

### 4.2. Intraspecific genetic diversity in non*-Saccharomyces*

Non-*Saccharomyces* excreted enzymes which have an impact on the technological traits are species and strain dependent. Therefore, it is necessary to discriminate each isolate before characterizing its potential as a producer or starter for industrial application. Although the discrimination within *S. cerevisiae* species was well reported by interdelta method and microsatellites, there are limited studies available for different non-*Saccharomyces* strains. In this study, we used TRtRNA to discriminate the non-*Saccharomyces* yeast strains. 34 and 40 distinct TRtRNA profiles were found using 5CAG and ISSR-MB, respectively. The primer ISSR-MB showed higher discriminatory capacity than primer 5CAG, as was also observed previously (Barquet et al., 2012; Nisiotou et al., 2022). This study achieved a lower discrimination power within *H. uvarum* and *M. pulcherrima* isolates. Four *M. pulcherrima* strains out of 22 strains were discriminated in this study using TRtRNA PCR method. The degree of variability (18.18%) was low compared to previous study (70.59%,12/17) (Barquet et al., 2012), which possibly due to the low diversity of this species in Ningxia.

### 4.3. Evaluation the colonizing of commercial non*-Saccharomyces* yeasts

The application of active dry yeast in wine fermentation is very common in the winemaking industry in China. Currently, Angel yeast NS-D (*T. delbrueckii*), Excellence B-Nature (*M. pulcherrima*) and Excellence X-Fresh (*L.thermotolerans*) were commonly used in Ningxia for increasing the complexity of the wines. Fortunately, the commercial non-*Saccharomyces* yeast strains were not detected during all the spontaneous fermentations in this study. However, it has been reported that commercial *S. cerevisiae* RC212 colonizing of the winery in Ningxia (Sun et al., 2017). It is understandable that the results could have contributed to the utiliation of Lalvin RC212 by local winery for more than 20 years, while the commercial non-*Saccharomyces* has been used for wine production in recent years. The application of active dry yeast in wine fermentation could reduce the diversity of indigenous yeasts and affect the regional characteristics of wines. Ningxia is one of the best known viticulture regions in China. Therefore, maintaining the biodiversity and preserving the genetic resources of indigenous non-*Saccharomyces* yeasts have become an urgent step in this region.

## 5. Conclusion

This study investigated the genetic diversity of non-*Saccharomyces* yeast at both species and strain level in five sub-regions of Ningxia, China, which has not previously been examined. The results of this study showed that different non-*Saccharomyces* strains were associated with different sub-regions. TRtRNA method is inadequate to discriminate species of *F. elegans*, *Filobasidium* sp., *M. pimensis* and *N. albida*. In addition, pairs of primers TRtNASC/ISSR-MB resulted in a higher discrimination power of *H. uvarum*, *C. zemplinina, M. pulcherrima, Cryptococcus sp.* and *R. glutinis* compared to TRtNASC/5CAG. This study is an important step before embarking on more time-consuming and labor-intensive methods to determine their performance in technological applications of interest. Their enological characteristics and interaction with *S. cerevisiae* worth further investigation.

## Data availability statement

The datasets presented in this study can be found in online repositories. The names of the repository/repositories and accession number(s) can be found in the article/Supplementary material.

## Author contributions

RL: Conceptualization, Formal analysis, Writing-original draft, Writing - review & editing. DF: Formal analysis, Investigation, Methodology, Writing – review & editing. HW: Formal analysis, Writing - review & editing. ZZ: Experiment, Data Curation, Writing - review & editing. NL: Data curation, Formal analysis, Writing-original draft. YS: Conceptualization, Funding acquisition, Methodology, Supervision, Writing - review & editing.

## Funding

This study was financially supported by the Key Research and Development Project of Ningxia Hui Autonomous Region (2022BBF02015, 2021BEF02014), National Natural Science Foundation of China (31960473), and Innovation project for Graduate Students of Ningxia University (GIP2021042).

## Conflict of interest

The authors declare that the research was conducted in the absence of any commercial or financial relationships that could be construed as a potential conflict of interest.

## Publisher’s note

All claims expressed in this article are solely those of the authors and do not necessarily represent those of their affiliated organizations, or those of the publisher, the editors and the reviewers. Any product that may be evaluated in this article, or claim that may be made by its manufacturer, is not guaranteed or endorsed by the publisher.

## Footnotes

^1^ http://www.ncbi.nlm.nih.gov/blast

## Abbreviations

TRtRNA: tandem repeat-tRNA.
