# Supplementary material for: Genetic diversity of non-Saccharomyces yeasts associated with spontaneous fermentation of Cabernet Sauvignon wines from Ningxia, China
Source: Front Microbiol. 2023 Aug 17;14:1253969. doi: 10.3389/fmicb.2023.1253969 (PMC10469914; doi:10.3389/fmicb.2023.1253969)
Supplement: Supplementary file 1 [file Data_Sheet_1.docx]

>Seq1 [organism= Aureobasidium pullulans] 26S rDNA D1/D2,partial cds of NXU 21-01 OP445815

ACTTCAGGATTGATAGTACGGCGAGTGAGCGGCAACAGCTCAAATTTGAAAGCTAGCCTTCGGGTTCGCATTGTAATTTGTAGAGGATGATTTGGGGAAGCCGCCTGTCTAAGTTCCTTGGAACAGGACGTCATAGAGGGTGAGAATCCCGTATGTGACAGGAAATGGCACCCTATGTAAATCTCCTTCGACGAGTCGAGTTGTTTGGGAATGCAGCTCTAAATGGGAGGTAAATTTCTTCTAAAGCTAAATATTGGCGAGAGACCGATAGCGCACAAGTAGAGTGATCGAAAGATGAAAAGCACTTTGGAAAGAGAGTTAAAAAGCACGTGAAATTGTTGAAAGGGAAGCGCTTGCAATCAGACTTGTTTAAACTGTTCGGCCGGTCTTCTGACCGGTTTACTCAGTTTGGACAGGCCAGCATCAGTTTCGGCGGCCGGATAAAGGCTCTGGGAATGTGGCCTCCACTTCGGTGGAGGTGTTATAGCCCAGGGTGTAATACGGCCAGCCGGGACTGAGGTCCGCGCTTCGGCTAGGATGCTGGCGTAATGGTTGTAAGCGACCCGTCTTGACCCCCGGACACCAAC

>Seq2 [organism= Cryptococcus albidus] 26S rDNA D1/D2,partial cds of NXU 21-02 OP445816

ATAATTAAGGATCCCCTAGTACGGCGAGTGAGCGGGAAGAGCTCAAATTTGAAATCTGGTAGCCTTCGGTTGCCCGAGTTGTAATCTAGAGAAGTGTTTTCCGTGCCGGCCCATGTACAAGTCCCTTGGAACAGGGCGTCATAGAGGGTGAGAATCCCGTCCTTGACATGGACCCCCGGTGCTTTGTGATACACTTTCAACGAGTCGAGTTGTTTGGGAATGCAGCTCAAAATGGGTGGTGAATTCCATCTAAAGCTAAATATTGGCGAGAGACCGATAGCGAACAAGTACCGTGAGGGAAAGATGAAAAGCACTTTGGAAAGAGAGTTAAACAGTACGTGAAATTGTTGAAAGGGAAACGATTGAAGTCAGTCATGCTCTTGGGACTTACCTCCCTTGAGTGGGGTCAACATCAGTTTTGATCGATGGATAAAGGCACGGGGAAGGTAGCACTCTCGGGTGAACTTATAGCCTCGCGTCATATACATTGATTGGGACTGAGGAACGCAGCATGCCTTATGGCCGGGATTCGTCCACGTACATGCTTAGGATGTTGACATAATGGCTTTAAACGACCCGTCTTG

>Seq3 [organism= Cryptococcus flavescens] 26S rDNA D1/D2,partial cds of NXU 21-03 OP445817

CCTCCGGGCATTCCCCTAGTACGGCGAGTGACCGGGAAGAGCTCAAATTTGAAATCTGGCGTGCTCAGTGCGTCCGAGTTGTAATCTATAGAGTCGTTTTCCGTGCCGGACTGTGTCCAAGTCCCTTGGAACAGGGTATCAAAGAGGGTGATAATCCCGTACTTGACACAATGACCGGTGCTCTGTGATACGTCTTCTACGAGTCGAGTTGTTTGGGAATGCAGCTCAAAATGGGTGGTGAGTTCCATCTAAAGCTAAATATTGGCGAGAGACCGATAGCGAACAAGTACCGTGAGGGAAAGATGAAAAGCACTTTGGAAAGAGAGTTAAACAGTACGTGAAATTGTTAAAAGGGAAACGATTGAAGTCAGTCGTGACTGAGAGGCTCAGCCGGTTCTGCCGGTGTATTCCCCTCAGTCGGGTCAACATCAGTTTTGTTCGGTGGATAAGGGCAGTTGGAAGGTGGCACCCTCGGGTGTGTTATAGCCAGCTGTCGCATACATCGGATGAGACTGAGGAATGCAGCTCGCCTTTATGGCCGGGGTTCGCCCACGTTCGAGCTTAGGATGTTGACATAATGGCTTTAAACGACCCGTCTTGACC

>Seq4 [organism= Cryptococcus terrestris] 26S rDNA D1/D2,partial cds of NXU 21-04 OP445818

AATCAAGGCATTCCCTAGTACGGCGAGTGACCGGGAAGAGCTCAAATTTGAAATCTGGCGTGCTCAGTGCGTCCGAGTTGTAATCTATAGAGGCGTTTTCCGTGCCGGACTGTGTCTAAGTCCCTTGGAACAGGGTATCAAAGAGGGTGATAATCCCGTACTTGACACAATGACCGGTGCTCTGTGATACGTCTTCTACGAGTCGAGTTGTTTGGGAATGCAGCTCAAAATGGGTGGTGAGTTCCATCTAAAGCTAAATATTGGCGAGAGACCGATAGCGAACAAGTACCGTGAGGGAAAGATGAAAAGCACTTTGGAAAGAGAGTTAAACAGTACGTGAAATTGTTGAAAGGGAAACGATTGAAGTCAGTCGTGACTGAGAGGCTCAGCCGGTTCTGCCGGTGTATTCCCCTCAGTCGGGTCAACATCAGTTTTGTTCGGTGGATAAGGGCAGCTGGAAGGTGGCACCTCCGGGTGTGTTATAGCCAGCTGTCGCATACACCGAATGAGACTGAGGAATGCAGCTCGCCTTTATGGCCGGGGTTCGCCCACGTCCGAGCTTAGGATGTTGACATAATGGCTTTAAACGACCCGTCTG

>Seq5 [organism= Cryptococcus magnus] 26S rDNA D1/D2,partial cds of NXU 21-05 OP445819

TTTCGAGGGATTCCCTAGTACGGCGAGTGAAGCGGGAAGAGCTCAAATTTGAAATCTGGTGGCCTCAGGTCATCCGAGTTGTAATCTATAGAAGTGTTTTCCGTGCTGGCTCATGTACAAGTCCCTTGGAACAGGGCGTCATAGAGGGTGAGAATCCCGTCCTTGACATGAACTACCAGTGCTCTGTGATACATTTTCAACGAGTCGAGTTGTTTGGGAATGCAGCTCAAAATGGGTGGTAAATTCCATCTAAAGCTAAATATTGGCGAGAGACCGATAGCGAACAAGTACCGTGAGGGAAAGATGAAAAGCACTTTGGAAAGAGAGTTAAACAGTATGTGAAATTGTTGAAAGGGAAACGATTGAAGTCAGTCGTGCTCTTTGGATTCAGCCGGTTCTGCCGGTGTACTTCCTTTGAGTGGGGTCAACATCAGTTTTGATCGCTGGATAAAGGCTGGAGGAACGTAGTACCCTCGGGTAAACTTATAGCCTCCTGTCACATACAGTGGTTGGGACTGAGGAACGCAGCACGCCTTTATGGCCGGGATTCGTCCACGTACGTGCTTAGGATGTTGACATAATGGCTTTAAACGACCCGTCTGA

>Seq6 [organism= Cystofilobasidium ferigula] 26S rDNA D1/D2,partial cds of NXU 21-06 OP445820

CCTTCGGGCATTCCCTAGTACGGCGAGTGAGCGGGAAAAGCTCAAATTTAAAATCTGGCAGTCTACGATTGTCCGAATTGTAATCTCGAGAAGTGTTTTCCGCGTTGGCCTGTGCACAAGTCCCTTGGAACAGGGCGTCATAGAGGGTGAGAATCCCGTCCTTGGCACAGACACCCAATGCTTTGTGATACACTCTCAATGAGTCGAGTTGTTTGGGAATGCAGCTCAAAATGGGAGGTAAATTCCTTCTAAAGCTAAATACTGGCGAGAGACCGATAGCGAACAAGTACCGTGAGGGAAAGATGAAAAGCACTTTGGAAAGAGAGTCAAACAGTACGTGAAATTGTTGAAAGGGAAACGATTGAAGTCAGTCGTGCTAGCCTGGATCCAGCCTTATGGTGTATCTCCAGGTCGGCAGGTCAGCATCAGTTTGGGAGGGTTAACAAGGGAGTTAGGAATGTGGCAACCTCGGTTGTGTTATAGCCTAGCTTCGCATTGATCCTGCTGGACTGAGGAACGCAGTGCGCCCGCAAGGGTTGGTCTTCGGACACATTCGCACTTAGGATGCTGGCATAATGGCTTTAAACGACCCGTCTTGACC

>Seq7 [organism= Candida zemplinina] 26S rDNA D1/D2,partial cds of NXU 21-07 OP445821

ACTTCAGGCATTGACTAGTACGGCGAGTGACAGGCAAGAGCTCAGATTTGAAAGGCACTTTTGTGCCGTTGTATTCTGAAGTTAGGGTCCTGAGAAACGATGCTTAAGTCTTCTGGAAAGGAGCGCCATGGAGGGTGATAGCCCCGTCTAGCATTGACCTCATATAGGATCTTAACATGGAGTCGAGTTGTTTGGGAATGCAGCTCAAATGGGTGGTATGCTCCATCTAAAGCTAAATATCTGCGAGAGACCGATAGTAAACAAGTACTGTGAGGGAAAGATGAAAAGAACTTTGAAAAGAGAGTGAAAAAGTACGTGAAATTGTTGAAATGGAAGGGTAGGCCGCTAACCATGTAGAGCCGTGTTTGGGGGGAAGATAAATGCTGTAGAATGTAGCTCCTCGGAGTATTATAGATGCAGTTCATATTCCCACCCGAGCGCGAGGATCTCAGGTTCTACTAAATGGTGGTCTACCACCCGTCTTGACCCCCGGACCC

>Seq8 [organism= Filobasidium magnum] 26S rDNA D1/D2,partial cds of NXU 21-08 OP445822

ATACGGGGATTCCCTAGTACGGCGAGTGAGCGGGAAGAGCTCAAATTTGAAATCTGGTGGCCTCAGGTCATCCGAGTTGTAATCTATAGAAGTGTTTTCCGTGCTGGCTCATGTACAAGTCCCTTGGAACAGGGCGTCATAGAGGGTGAGAATCCCGTCCTTGACATGAACTACCAGTGCTCTGTGATACATTTTCAACGAGTCGAGTTGTTTGGGAATGCAGCTCAAAATGGGTGGTAAATTCCATCTAAAGCTAAATATTGGCGAGAGACCGATAGCGAACAAGTACCGTGAGGGAAAGATGAAAAGCACTTTGGAAAGAGAGTTAAACAGTATGTGAAATTGTTGAAAGGGAAACGATTGAAGTCAGTCGTGCTCTTTGGATTCAGCCGGTTCTGCCGGTGTACTTCCTTTGAGTGGGGTCAACATCAGTTTTGATCGCTGGATAAAGGCTGGAGGAACGTAGTACCCTCGGGTAAACTTATAGCCTCCTGTCACATACAGTGGTTGGGACTGAGGAACGCAGCACGCCTTTATGGCCGGGATTCGTCCACGTACGTGCTTAGGATGTTGACATAATGGCTTTAAACGACCCGTCTA

>Seq9 [organism= Filobasidium elegans] 26S rDNA D1/D2,partial cds of NXU 21-09 OP445823

ACATAAGGATTCCCTAGTACGGCGAGTGAGCGGGAAGAGCTCAAATTTGAAATCTGGTGGCCTCAGGTCATCCGAGTTGTAATCTATAGAAGTGTTTTCCGTGCTGGCTCATGTACAAGTCCCTTGGAACAGGGCGTCATAGAGGGTGAGAATCCCGTCCTTGACATGAACTACCAGTGCTCTGTGATACATTTTCAACGAGTCGAGTTGTTTGGGAATGCAGCTCAAAATGGGTGGTAAATTCCATCTAAAGCTAAATATTGGCGAGAGACCGATAGCGAACAAGTACCGTGAGGGAAAGATGAAAAGCACTTTGGAAAGAGAGTTAAACAGTATGTGAAATTGTTGAAAGGGAAACGATTGAAGTCAGTCGTGCTCTTTGGATTCAGCCGGTTCTGCCGGTGTACTTCCTTTGAGTGGGGTCAACATCAGTTTTGATCGCTGGATAAAGGCTGGAGGAACGTAGTACCCTCGGGTAAACTTATAGCCTCCTGTCACATACAGTGGTTGGGACTGAGGAACGCAGCACGCCTTTATGGCCGGGATTCGTCCACGTACGTGCTTAGGATGTTGACATAATGGCTTTAAACGACCCGTCTGA

>Seq10 [organism= Hanseniaspora uvarum] 26S rDNA D1/D2,partial cds of NXU 21-10 OP445824

ACTCAGGGATACTTAGTACGGCGAGTGAGCGGTAAAAGCTCAAATTTGAAATCTGGTACTTTCAGTGCCCGAGTTGTAATTTGTAGAATTTGTCTTTGATTAGGTCCTTGTCTATGTTCCTTGGAACAGGACGTCATAGAGGGTGAGAATCCCGTTTGGCGAGGATACCTTTTCTCTGTAAGACTTTTTCGAAGAGTCGAGTTGTTTGGGAATGCAGCTCAAAGTGGGTGGTAAATTCCATCTAAAGCTAAATATTGGCGAGAGACCGATAGCGAACAAGTACAGTGATGGAAAGATGAAAAGAACTTTGAAAAGAGAGTGAAAAAGTACGTGAAATTGTTGAAAGGGAAGGGCATTTGATCAGACATGGTGTTTTTTGCATGCACTCGCCTCTCGTGGGCTTGGGCCTCTCAAAAATTTCACTGGGCCAACATCAATTCTGGCAGCAGGATAAATCATTAAGAATGTAGCTACTTCGGTAGTGTTATAGCTTTTTGGAATACTGTTAGCCGGGATTGAGGACTGCGCTTCGGCAAGGATGTTGGCATAATGGTTAAATGCCGCCCGTCTTG

>Seq11 [organism= Metschnikowia pimensis] 26S rDNA D1/D2,partial cds of NXU 21-11 OP445825

ACTCGGGCATGCCAGTACGGCGAGTGAGCGGCAAAAGCTCAAATTTGAAATCCTCAGGGAATTGTAATTTGAAGATGTTTGGGTCAAGGCTAGCAGAGGTTAAGTCCACTGGAACGTGGCGCCACAGAGGGTGACAGCCCCGTGAACCTCCTAAAGCCCCTTACCCCAAACCTCCAAGAGTCGAGTTGTTTGGGAATGCAGCTCTAAGTGGGTGGTAAATTCCATCTAAAGCTAAATACCGGCGAGAGACCGATAGCGAACAAGTACAGTGATGGAAAGATGAAAAGCACTTTGAAAAGAGAGTGAAAAAGTACGTGAAATTGTTGAAAGGGAAGGGCTTGCAAGCAGACACTTAATTGGGCCAGCATCGGGGCGGCAGGGAGAAAACCTCCCGGGAAATGTACCTCTCGAGTGTTATAGTCCCGGGCATCCTCTCCCATTCTGCCCCGAGGCCTGCGTATCTAGGATGCTGGCGTAATGGTTGCAAGTCGCCCGTCTTG

>Seq12 [organism= Metschnikowia pulcherrima] 26S rDNA D1/D2,partial cds of NXU 21-12 OP445826

ACAGGGGCGTTGCTCAGTACGGCGAGTGAGCGGCAAAAGCTCAAATTTGAAATCCCCCGGGAATTGTAATTTGAAGAGATTTGGGTCCGGCCGGCGGGGGTTAAGTCCACTGGAAAGTGGCGCCACAGAGGGTGACAGCCCCGTGAACCCCTTCAACGCCCTCATCCCAGATCTCCAAGAGTCGAGTTGTTTGGGAATGCAGCTCTAAGTGGGTGGTAAATTCCATCTAAAGCTAAATACCGGCGAGAGACCGATAGCGAACAAGTACAGTGATGGAAAGATGAAAAGCACTTTGAAAAGAGAGTGAAAAAGTACGTGAAATTGTTGAAAGGGAAGGGCTTGCAAGCAGACACTTAACTGGGCCAGCATCGGGGCGGCGGGAAGCAAAACCACCGGGGAATGTACCTTTCGAGGATTATAACCCCGGTCCTTACTTCCACACCACCCCGAGGCCTGCAATCTAAGGATGCTGGCGTAATGGTTGCAAGTCGCCCGTCTTGAACACGGACCA

>Seq13 [organism= Naganishia albida] 26S rDNA D1/D2,partial cds of NXU 21-13 OP445827

ATTTAAGGATTCCCTAGTACGGCGAGTGAGCGGGAAGAGCTCAAATTTGAAATCTGGTAGCCTTCGGTTGCCCGAGTTGTAATCTAGAGAAGTGTTTTCCGTGCCGGCCCATGTACAAGTCCCTTGGAACAGGGCGTCATAGAGGGTGAGAATCCCGTCCTTGACATGGACCCCCGGTGCTTTGTGATACACTTTCAACGAGTCGAGTTGTTTGGGAATGCAGCTCAAAATGGGTGGTGAATTCCATCTAAAGCTAAATATTGGCGAGAGACCGATAGCGAACAAGTACCGTGAGGGAAAGATGAAAAGCACTTTGGAAAGAGAGTTAAACAGTACGTGAAATTGTTGAAAGGGAAACGATTGAAGTCAGTCATGCTCTTGGGACTTACCTCCCTTGAGTGGGGTCAACATCAGTTTTGATCGATGGATAAAGGCACGGGGAAGGTAGCACTCTCGGGTGAACTTATAGCCTCGCGTCATATACATTGATTGGGACTGAGGAACGCAGCATGCCTTATGGCCGGGATTCGTCCACGTACATGCTTAGGATGTTGACATAATGGCTTTAAACGACCCGTCTG

>Seq14 [organism= Pichia kluyveri] 26S rDNA D1/D2,partial cds of NXU 21-14

OP445828

TTAGGCGCTGGCATGCCTCAGTAGCGGCGAGTGAGCGGCAAGAGCTCAGATTTGAAATCTCACCTAGTGTGCGAGTTGTAAATTGCAGGTTGGAGTCTCGGGTTAGACGTGTGTGCAAGTCCCTTGGAACAGGGTGCCACTGAGGGTGAGAGCCCCGTAGCGTGCATGTCGACACCTGTGAGGCCCTTCTGACGAGTCGAGTTGTTTGGGAATGCAGCTCTAAGTGGGTGGTAAATTCCATCTAAGGCTAAATATTGGCGAGAGACCGATAGCGAACAAGTACTGTGAAGGAAAGATGAAAAGCACTTTGAAAAGAGAGTGAAACAGCACGTGAAATTGTTGAAAGGGAAGGGTATTGGGCTCGACATGGGATTTACGCATCGTTGCCTCTCGTGGGCGGCGCTCTGGGTTTTTCCTGGGCCAGCATCGGTTTTCGTTGCAGGATAAGGACAATTGGAATGTGGCTCCTCGGAGTGTTATAGCCTTTTGTAGATGCTGCGTATGGGGACCGAGGGCTGCGGCGGACTCGTTTCGTCTCGGATGCTGGCACAACGGCGCAATACCGCCCGTCTTGAAACACGGACCA

>Seq15 [organism= Pichia kudriavzevii] 26S rDNA D1/D2,partial cds of NXU 21-15 OP445829

ACAAAGGGCATTGCTCAGTAGCGGCGAGTGAGCGGCAAGAGCTCAGATTTGAAATCTCACCTAGTGTGCGAGTTGTAAATTGCAGGTTGGAGTCTCGGGTTAGACGTGTGTGCAAGTCCCTTGGAACAGGGCGCCACTGAGGGTGAGAGCCCCGTATCGTGCATGTCGACACCTGTGAGGCCCTTCTGACGAGTCGAGTTGTTTGGGAATGCAGCTCTAAGTGGGTGGTAAATTCCATCTAAGGCTAAATATTGGCGAGAGACCGATAGCGAACAAGTACTGTGAAGGAAAGATGAAAAGCACTTTGAAAAGAGAGTGAAACAGCACGTGAAATTGTTGAAAGGGAAGGGTATTGGGCTCGACATGGGATTTACGCATCGTTGCCTCTCGTGGGCGGCGCTCTGGGTTTTTCCTGGGCCAGCATCGGTTTTCGTTGCAGGATAAGGACAATTGGAATGTGGCTCCTCGGAGTGTTATAGCCTTTTGTAGATGCTGCGTATGGGGACCGAGGGCTGCGGCGGACTCGTTTCGTCTCGGATGCTGGCACAACGGCGCAATACCGCCCGTCTTGAACCGGGGACAACA

>Seq16 [organism= Rhodotorula glutinis] 26S rDNA D1/D2,partial cds of NXU 21-16 OP445830

AATCGGGCATTCCCTAGTAGCGGCGAGCGAAGCGGGAAGAGCTCAAATTTATAATCTGGCACCTTCGGTGTCCGAGTTGTAATCTCTAGAAGTGTTTTCCGCGTTGGACCGCACACAAGTCTGTTGGAATACAGCGGCATAGTGGTGAAACCCCCGTATATGGTGCGGACGCCCAGCGCTTTGTGATACACTTTCAATGAGTCGAGTTGTTTGGGAATGCAGCTCAAATTGGGTGGTAAATTCCATCTAAAGCTAAATATTGGCGAGAGACCGATAGCGAACAAGTACCGTGAGGGAAAGATGAAAAGCACTTTGGAAAGAGAGTTAACAGTACGTGAAATTGTTGGAAGGGAAACGCTTGAAGTCAGACTTGCTTGCCGGAGCTTGCTTCGGTTTGCAGGCCAGCATCAGTTTTCCGGGGTGGATAATGACGGTTTGAAGGTAGCAGTCTCGGCTGTGTTATAGCTTTCCGTTGGATACATCCTGGGGGACTGAGGAACGCAGCGTGCTTTTTGCGAAAGACTCGTCTTTTTCACGCTTAGGATGCTGGTGGAATGGCTTTAAACGACCCGTCTTAA

>Seq17 [organism= Rhodotorula graminis] 26S rDNA D1/D2,partial cds of NXU 21-17 OP445831

TTTCTAAGGATGCCTAGTAGCGGCGAGCGAGCGGGAGAGCTCAAATTTATAATCTGGCACCTTCGGTGTCCGAGTTGTAATCTCTAGAAGTGTTTTCCGCGTTGGACCGCACACAAGTCTGTTGGAATACAGCGGCATAGTGGTGAAACCCCCGTATATGGTGCGGACGCCCAGCGCTTTGTGATACACTTTCAATGAGTCGAGTTGTTTGGGAATGCAGCTCAAATTGGGTGGTAAATTCCATCTAAAGCTAAATATTGGCGAGAGACCGATAGCGAACAAGTACCGTGAGGGAAAGATGAAAAGCACTTTGGAAAGAGAGTTAACAGTACGTGAAATTGTTGGAAGGGAAACGCTTGAAGTCAGACTTGCTTGCCGGAGCTTGCTTCGGTTTGCAGGCCAGCATCAGTTTTCCGGGGTGGATAATGACGGTTTGAAGGTAGCAGTCTCGGCTGTGTTATAGCTTTCCGTTGGATACATCCTGGGGGACTGAGGAACGCAGCGTGCTTTTTGCGAAAGACTCGTCTTTTTCACGCTTAGGATGCTGGTGGAATGGCTTTAAACGACCCGTCTTGA

>Seq18 [organism= Cryptococcus sp.] 26S rDNA D1/D2,partial cds of NXU 21-18

OQ857299

ATATAAGGGCATTCCCTAGTACGGCGAGTGAAGCGGGAAGAGCTCAAATTTGAAATCTGGTAGCCTTCGGTTGCCCGAGTTGTAATCTAGAGAAGTGTTTTCCGCGTTGGCCCATGTACAAGTCCCTTGGAACAGGGCGTCATAGAGGGTGAGAATCCCGTCCTTGACATGGACCCCCAATGCTTTGTGATACACTTTCAACGAGTCGAGTTGTTTGGGAATGCAGCTCAAAATGGGTGGTGAATTCCATCTAAAGCTAAATATTGGCGAGAGACCGATAGCGAACAAGTACCGTGAGGGAAAGATGAAAAGCACTTTGGAAAGAGAGTTAAACAGTACGTGAAATTGTTGAAAGGGAAACGATTGAAGTCAGTCATGCTCTTTGGATTAAGCCGTTCTGCGGTGTATTTCATTGAGCGGGGTCAACATCAGTTTTGATCGCTGGAAAAGGGCAGGAGGAAGGTAGCACTCTCGGGTGAACTTATAGCCTCTTGTCGTATACAGTGATTGGGACTGAGGAACGCAGCATGCCTTTTGGCCGGGATTCGTCCACGTACATGCTTAGGATGTTGACATAATGGCTTTAAACGACCCGTCTTAA

>Seq19 [organism= Filobasidium sp.] 26S rDNA D1/D2,partial cds of NXU 21-19

OQ857300

CTTAGGGGATCCCTAGTACGGCGAGTGAGCGGGAAGAGCTCAAATTTGAAATCTGGTGGCCTCAGGTCATCCGAGTTGTAATCTATAGAAGTGTTTTCCGTGCTGGCTCATGTACAAGTCCCTTGGAACAGGGCGTCATAGAGGGTGAGAATCCCGTCCTTGACATGAACTACCAGTGCTCTGTGATACATTTTCAACGAGTCGAGTTGTTTGGGAATGCAGCTCAAAATGGGTGGTAAATTCCATCTAAAGCTAAATATTGGCGAGAGACCGATAGCGAACAAGTACCGTGAGGGAAAGATGAAAAGCACTTTGGAAAGAGAGTTAAACAGTATGTGAAATTGTTGAAAGGGAAACGATTGAAGTCAGTCGTGCTCTTTGGATTCAGCCGGTTCTGCCGGTGTACTTCCTTTGAGTGGGGTCAACATCAGTTTTGATCGCTGGATAAAGGCTGGAGGAACGTAGTACCCTCGGGTAAACTTATAGCCTCCTGTCACATACAGTGGTTGGGACTGAGGAACGCAGCACGCCTTTATGGCCGGGATTCGTCCACGTACGTGCTTAGGATGTTGACATAATGGCTTTAAACGACCCGTCTG
