# Supplementary figures and images for: Genetic diversity of non-Saccharomyces yeasts associated with spontaneous fermentation of Cabernet Sauvignon wines from Ningxia, China
Source: Front Microbiol. 2023 Aug 17;14:1253969. doi: 10.3389/fmicb.2023.1253969 (PMC10469914; doi:10.3389/fmicb.2023.1253969)

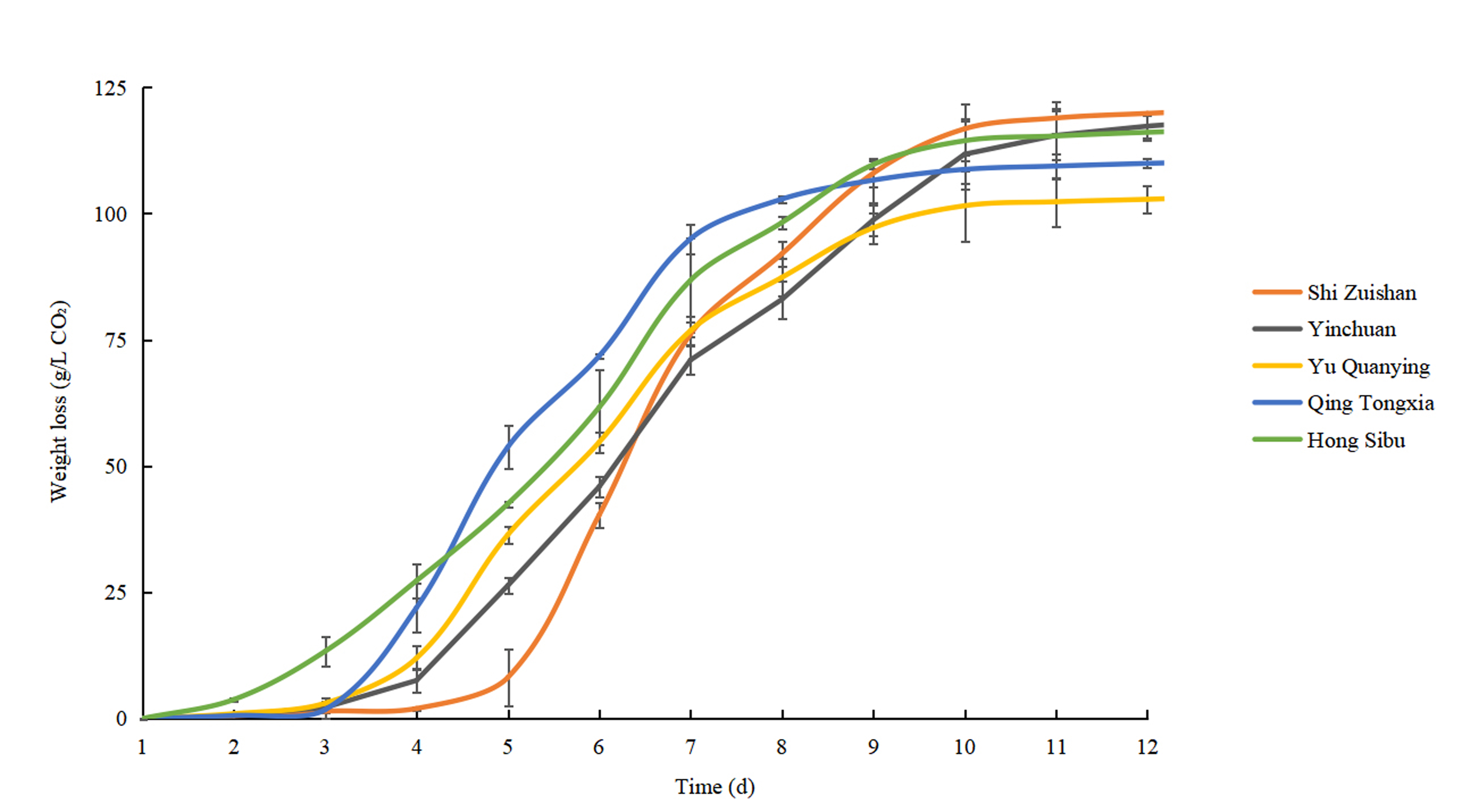

Supplement: Supplementary file 2 [file Image_1.JPEG]
