# Supplementary material for: Genetic diversity of non-Saccharomyces yeasts associated with spontaneous fermentation of Cabernet Sauvignon wines from Ningxia, China
Source: Front Microbiol. 2023 Aug 17;14:1253969. doi: 10.3389/fmicb.2023.1253969 (PMC10469914; doi:10.3389/fmicb.2023.1253969)
Supplement: Supplementary file 3 [file Table_1.docx]

Supplementary Table S1

| Sub-regions | Grapes* | | | Wines | |
| --- | --- | --- | --- | --- | --- |
|  | Total Sugar (g/L) | Total Acids (g/L) | pH | Alcohol (%Vol) | Residual Sugars (g/L) |
| Shi Zuishan | 250.70 ± 1.10 | 4.40 ± 0.00 | 3.54 ± 0.00 | 15.00 ± 0.09 | 2.30 ± 0.01 |
| Yinchuan | 239.10 ± 0.26 | 4.30 ± 0.00 | 3.50 ± 0.00 | 15.00 ± 0.10 | 3.80 ± 0.05 |
| Yu Quanying | 210.47 ± 0.06 | 5.60 ± 0.00 | 3.30 ± 0.01 | 13.30 ± 0.06 | 2.52 ± 0.05 |
| Qin Tongxia | 226.60 ± 0.36 | 4.40 ± 0.00 | 3.55 ± 0.00 | 13.90 ± 0.03 | 3.10 ± 0.04 |
| Hong Sibu | 241.50 ± 0.10 | 5.60 ± 0.00 | 3.33 ± 0.00 | 15.10 ± 0.17 | 2.50 ± 0.02 |

* Data of sugar content, total acidity and pH of grapes in Yinchuan, Yu Quanying, Qing Tongxia were shown according to Zhang et al. (2022).

**References**

Zhang, Z., Zhang, Q. C., Yang, H., Sun, L. J., Xia, H. C., and Sun, W. J., et al. (2022). Bacterial Communities Related to Aroma Formation during Spontaneous Fermentation of ‘Cabernet Sauvignon’ Wine in Ningxia, China. *Foods*. 11(18), 2775. doi: 10.3390/foods11182775
